# A survey of severe acute respiratory coronavirus virus 2 (SARS-CoV-2)–positive healthcare providers: Household prevention measures and stress early in the pandemic

**DOI:** 10.1017/ash.2021.221

**Published:** 2021-11-26

**Authors:** Sebastian Otero, Leena B. Mithal, Anum I. Khan, Antonia S. Willnow, Ami B. Patel, Mehreen Arshad

**Affiliations:** 1 Division of Infectious Diseases, Ann & Robert H. Lurie Children’s Hospital, Chicago, Illinois, United States; 2 Department of Pediatrics, Northwestern University Feinberg School of Medicine, Chicago, Illinois, United States; 3 Institute of Health Policy, Management and Evaluation, University of Toronto, Ontario, Canada

## Abstract

Measures to prevent coronavirus disease 2019 (COVID-19) spread to household members was assessed by surveying COVID-19–positive physicians and advanced practice providers. Showering and changing were more common than physical distancing. Half of respondents reported a symptomatic household member. Most reported increased stress, worsening of mental health, and concerns about illness and impact on family.

Essential workers confronted much of the risk at the onset and throughout much of the severe acute respiratory syndrome coronavirus 2 (SARS-CoV-2) pandemic.^1,2^ Infection prevention measures for healthcare providers (HCPs) have focused on preventing hospital-acquired coronavirus disease 2019 (COVID-19).^3^ Much of the literature has centered around precautions needed in hospital settings and risk of acquisition of SARS-CoV-2 in these high-risk settings. Literature examining infection prevention measures to protect household members of HCPs is scarce,^4^ and well-being of self and family have been a prominent source of anxiety and stress for most HCPs.^5^ Recent anecdotal reports showed that HCPs practice a range of preventive measures including complete isolation from their family early in the pandemic.^6^ In this study, we identified infection prevention measures taken in the households of COVID-19–positive HCPs, analyzed their potential effectiveness in reducing symptomatic disease transmission, and explored the impact of stress in protective measures undertaken by HCPs.

## Methods

We conducted a cross-sectional survey of COVID-19–positive HCPs (physicians, advanced practice nurses, or physician assistants) between May and September 2020. Responses were collected through a REDCap survey that was accessible through an online link. This link was posted on HCP-only Facebook groups, e-mail listservs (hospital systems and academic departments at the discretion of administrative head), and institutional websites of medical centers. The survey was approved by the Lurie Children’s Hospital institutional review board and included a provision of consent. Responses were included in the analysis if they met the following criteria: the respondent (1) was an HCP in the United States, (2) had a confirmed or presumed diagnosis of COVID-19, and (3) had at least 1 other member in their household.

The survey comprised of 27 questions, both self-reported multiple choice and free-text options, including respondent demographic information, infection and transmission prevention measures both in hospital settings and in the home environment (referred to herein as “home prevention”), household demographic information and family members’ COVID-19 status and their symptoms, and the respondent’s self-reported stress/anxiety levels and sources of stress (survey available in a Supplementary File). The types of prevention measures used by HCPs were categorized into fomite decontamination (eg, changing clothing and/or showering), separation (eg, living in a separate room or living completely separate from family), and physical distancing measures (eg, refraining from physical contact, no intimate contact, wearing mask at home). Hospital measures were categorized as standard recommended precautions (ie, mask, gown, glove, eyewear), and additional precautions (ie, above standard recommendations such as face shield, hair covering and scrubs). Because it was not consistently possible to collect individual polymerase chain reaction (PCR) or serological test results within the scope of the survey, the COVID-19 status of household members was recorded as “symptomatic” or “not symptomatic.”

Bivariate analyses using Pearson χ^2^ and Fisher exact tests were conducted to explore the relationship between individual variables (ie, prevention measures, stress levels) and family member COVID-19 symptom status. Statistical analyses were conducted in STATA version 16.1 software (StataCorp, College Station, TX).

## Results

**Fig. 1.**
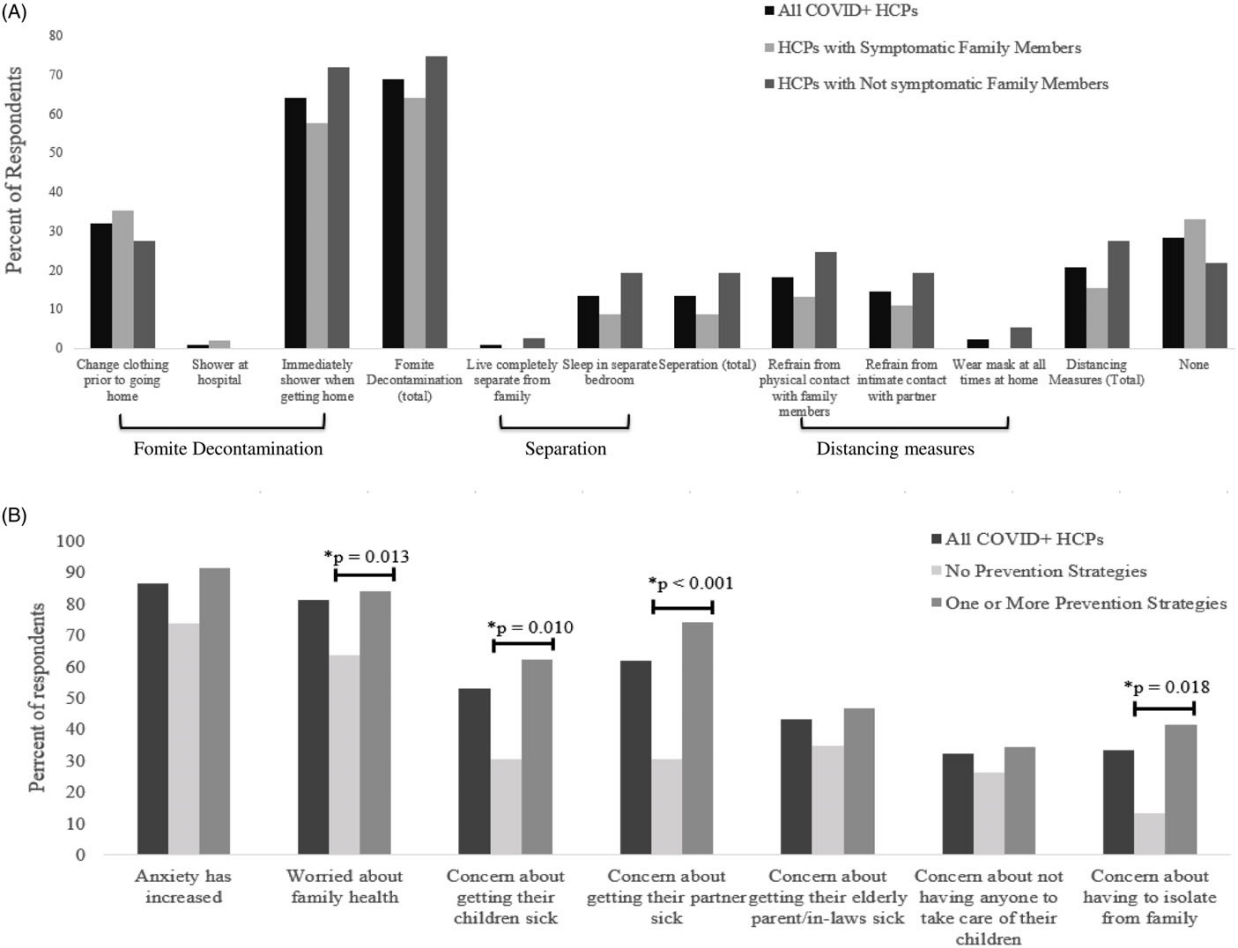
(A) Household infection prevention measures taken by COVID-19-positive healthcare providers. (B) Stress measures among healthcare providers and household prevention strategies.

Among the 81 HCPs surveyed, 58% worked in an outpatient setting, 26% worked in inpatient units, 6% worked in emergency departments, and 7% worked in an intensive care unit. Overall, 62% of respondents reported having mild COVID-19 symptoms and 37% had moderate symptoms; only 1 HCP required hospitalization. Sample demographic characteristics, COVID-19 symptoms, and household composition are summarized in Table 1. Moreover, 45 HCPs (56%) reported having symptomatic household members, although 36 (44%) reported having no symptomatic household members. Also, 23 respondents (28%) reported using no home prevention measures, 37 (46%) reported using 1 prevention measure, and 21 (26%) reported using multiple measures to prevent the spread of COVID-19 in their home (Fig. 1A). We detected no significant association between the use of certain in-hospital prevention measures and having symptomatic household members.

**Table 1.** Demographics and Household Members of COVID-19–Positive Healthcare Providers

Variable	All COVID-19+ HCPs (n=81), No. (%)	HCPs with Symptomatic Household Members (n=45), No. (%)	HCPs with No Symptomatic Household Members (n=36), No. (%)
Age, mean y (SD)	41.6 (8.8)	42.6 (8.9)	40.3 (8.6)
Sex, female	70 (86.4)	39 (86.7)	31 (86.1%)
Provides outpatient care	47 (58.0)	25 (55.6)	22 (61.1)
People in household, median (IQR)	3 (IQR 2)	3 (IQR 2)	3 (IQR 2)
COVID-19 test (PCR or serology)	70 (86.4)	35 (77.8)	35 (97.2)
**Symptoms of COVID-19 in HCP**			
Cough	51 (63.0)	32 (71.1)	19 (52.8)
Difficulty breathing	33 (40.7)	20 (44.4)	13 (36.1)
Fever	50 (61.7)	31 (68.9)	29 (52.8)
Abdominal pain	14 (17.3)	10 (22.2)	4 (11.1)
Vomiting or diarrhea	31 (38.3)	17 (37.8)	14 (38.9)
Rash	4 (5.0)	3 (6.7)	1 (2.8)
Anosmia	40 (49.4)	22 (48.9)	18 (50.0)
Malaise	64 (79.0)	38 (84.4)	26 (72.2)
**Age of household members**			
< 1 y	4 (5.0)	1 (2.2)	3 (8.3)
1–9 y	47 (58.0)	29 (64.4)	18 (50.0)
10–17 y	28 (32.1)	15 (33.3)	11 (30. 6)
18–64 y	80 (95.1)	43 (95.6)	34 (94.4)
≥65 y	10 (11.1)	6 (11.1)	4 (11.1)
**Age of household members with COVID-19 symptoms**			
<1 y		1 (2.3)	
1–9 y		18 (30.0)	
10–17 y		6 (13.3)	
18–64 y		40 (88.9)	
≥65 y		3 (6.8)	
**Relationship of household members with COVID-19 Symptoms**			
Partner		38 (84.4)	
Children		25 (55.6)	
Parent		1 (2.2)	
Grandparent		0	
Other family, same age		2 (4.4)	
Other family, younger		2 (4.4)	
Other family, older		0	

Fomite decontamination measures were the most common, with almost 70% of total respondents practicing these measures (Fig. 1A). Of the 23 HCPs surveyed who reported using no home prevention measures, 15 (65%) had symptomatic household members. Among the 58 HCPs using 1 or more prevention measures, 30 (52%) reported symptomatic household members. We noted a trend toward lower likelihood of having a symptomatic household member among HCPs taking multiple types of prevention measures compared to HCPs taking 1 or no prevention measures (*P* = .061). No single home prevention measure or prevention category was significantly associated with having a symptomatic household member.

The vast majority (86%) of HCPs reported an increase in their anxiety level, with 83% also reporting a moderate to significant worsening in their mental health, regardless of whether there were symptomatic household members (*P* = .94) (Fig. 1B). HCPs using 1 or more types of home prevention measures were more likely to report being worried about their family’s health (*P* = .013), getting their child(ren) sick (*P* = .01), getting their partner sick (*P* < .001), and concern of having to isolate from their family (*P* = .018). Showering and/or changing after work was associated with increase worry of family health (*P* = .005), while all other prevention measures were not significantly different based on reported stressors.

## Discussion

Although anecdotal evidence exists of the broad range of measures taken by HCPs to protect their families, this is the first study addressing this issue. Our survey results show that HCPs implemented a variety of infection prevention measures within their household environments. Fomite decontamination measures such as changing clothing and showering after work were practiced more frequently than physical distancing from household members, whereas ∼28% of HCPs took no targeted prevention measures at home.

Stress, anxiety, and the worsening of mental health was widely reported in COVID-19–positive HCP participants. In particular, concerns about making household members ill and impact on family were significant sources of stress. However, research with larger sample sizes are needed to better understand the dynamics between pandemic-related stress and the extent of infection prevention measures that HCPs are willing to take to keep their families safe. Despite vaccine roll-out across the country, the issue of household viral transmission continues to be a concern not only among HCPs but also nurses, other essential frontline workers, and teachers and/or childcare providers given a lag in vaccination of children. Future research and recommendations for home transmission-mitigation strategies must focus on balancing the mental health impact of lifestyle changes with protecting the health of high-risk workers and their families.

Our study was limited by the design and methods of recruitment, which affects generalizability to all frontline workers. Additionally, the small sample limits our ability to explore differences between groups in a comprehensive manner. We also did not have PCR or serologic data on household members to assess asymptomatic transmission. We did not consider the longitudinal change in prevention measures enacted within HCP households as new information about effective prevention measures for SARS-CoV-2 and vaccination emerged.

As global health agencies discuss lessons learnt from the current pandemic and debate strategies for future pandemic preparedness, more research is needed to better understand effective prevention measures of infections within households of HCPs and the influences of stress during novel infectious disease outbreaks. More broadly, the next steps on this research topic should consider including not only healthcare professionals and allied fields but all those deemed essential workers.
